# Contribution of Cross-sectional Imaging in the Work-up of Intermediate Coronary Artery Stenosis

**DOI:** 10.5334/jbsr.1537

**Published:** 2018-04-20

**Authors:** Olivier Ghekiere

**Affiliations:** 1 Jessa Ziekenhuis Hasselt and CHC Hospital Liege, BE

**Keywords:** Intermediate coronary artery stenosis, quantitative coronary CT angiography, non-invasive FFR estimates from CT (FFRCT), stress perfusion cardiac magnetic resonance (CMR), relative myocardial perfusion index, fractional flow reserve (FFR)

Adequate selection of hemodynamically significant lesions is important, as patients without ischemia-causing stenoses – representing approximately two-thirds of intermediate stenoses – do not benefit from revascularization [1]. On the contrary, they show favorable outcomes on optimal medical therapy alone. Catheter-based fractional flow reserve (FFR) measurement during coronary angiography remains the current standard in predicting the functional significance of lesion-specific coronary artery stenosis. In clinical practice, FFR values ≤ 0.80 identify inducible ischemia and require coronary revascularization [2,3,4].

The aim of this thesis was to evaluate how recent advances in non-invasive cardiac imaging, including quantitative coronary CT angiography (CTA), non-invasive FFR estimates from CT (FFR_CT_) and stress perfusion cardiac magnetic resonance (CMR) can represent an alternative in the management of intermediate coronary stenosis.

Using coronary CTA, we demonstrated that quantitative stenosis analysis and plaque descriptors poorly correlated with the functional significance of intermediate coronary stenosis, although significant correlations were obtained between coronary CTA and quantitative coronary angiography (QCA) for minimal lumen diameter and diameter stenosis percentage. Best predictors for FFR ≤ 0.80 coronary stenosis were: minimal lumen diameter ≤ 1.35 mm, and ≤ 2.3 mm^2^ minimal lumen area on quantitative coronary CTA and minimal lumen diameter ≤ 1.1 mm on quantitative coronary angiography [5]. These thresholds were similar to other studies [6,7] and higher than the minimal lumen diameter cut-off of 1.1 mm on QCA.

Technical and patient-related factors that may adversely affect coronary CTA image quality, and solutions to mitigate the impact of the image artifacts were discussed in a review paper. Recent technical evolutions in modern CT scanners have significantly improved coronary CTA image quality because of a larger scanning coverage, and higher temporal and spatial resolution. Easier acquisition, post-processing and better diagnostic confidence are expected from the ongoing image quality improvement. Additionally, patient safety has improved by dose-reduction strategies and iterative reconstruction algorithms, while the high quality of the images could be maintained [8,9]. Recent achievements indicate that even further dose reductions are to be expected in the near future [8].

When we used these technical advances with a latest generation 320-slice CT-scanner, non-diagnostic motion-related image quality was exceptional. This image quality was mainly influenced by the coronary diameter, while higher heart rates had less negative impact. Therefore, in order to increase the coronary diameter, the use of sublingual nitroglycerin is strongly advocated before scanning, and scan parameters should be adjusted to the patient’s heart rate to improve both image quality and diagnostic confidence [10]. Severe coronary calcifications remained a significant cause of image quality impairment, indicating that the current spatial resolution may still not be sufficient to visualize smaller coronary arteries with a diagnostic quality [11].

These findings confirm the poor relation between angiographic parameters and the functional severity of an intermediate-grade stenosis, even with the most recent technologies using quantitative coronary CTA. Therefore, revascularization of theses stenoses should be guided not only by anatomical estimation, but requires additional functional evaluation as well [2].

In the second part of this thesis, we investigated the assessment of intermediate coronary stenosis by stress perfusion CMR imaging and FFR_CT_.

Invasive FFR equals the ratio of the mean distal coronary to the mean aortic pressure during hyperemia. The concept of FFR was initially validated as a relative flow reserve, the ratio of hyperemic flow in a stenotic coronary artery to hyperemic flow in a normal coronary artery. Therefore it avoids the confounding effect of microvascular disease [12,13].

In the daily clinical practice, stress perfusion CMR considers stress or stress versus rest (myocardial perfusion reserve index) and is highly sensitive to microvascular disease [14]. Correcting the myocardial perfusion downstream a coronary stenosis (culprit) for perfusion in normal (non-culptrit) myocardium during hyperemia defines the relative myocardial perfusion index, which is potentially unaffected by microvascular obstruction. Our hypothesis was that assessing the relative myocardial perfusion index by stress perfusion CMR will enhance the lesion-specific correlation with FFR.

The relative myocardial perfusion index, including the correction for perfusion in normal myocardium (culprit/non-culprit), resulted in higher correlations with the FFR value than uncorrected perfusion in culprit myocardium (0.587 versus 0.273; p < 0.001) and the myocardial perfusion reserve index (0.587 versus 0.289; p < 0.001) in a study with 49 intermediate coronary stenoses. Using a cut-off value of 0.84 of the relative myocardial perfusion index revealed a high diagnostic accuracy (91% sensitivity and 80% specificity) to predict a FFR ≤ 0.80 intermediate coronary stenosis. These findings are supported by the correlations observed between the FFR and Positron Emission Tomography assessment of the functional significance of coronary artery stenoses in two recent studies [15,16]. These studies reported also the highest diagnostic accuracy to predict lesion-specific ischemia after correction of the myocardial blood flow by including remote myocardium compared to other quantitative assessment, such as stress myocardial blood flow and coronary flow reserve.

The relative myocardial perfusion index is more sensitive for lesion-specific ischemia than visual analysis as it is potentially unaffected by microvascular obstruction correction. The analysis of the relative myocardial perfusion index yielded an improved sensitivity of 91% (versus 73%) and overall diagnostic accuracy of 88% (versus 82%) to determine FFR ≤ 0.80 intermediate-grade stenosis compared to visual consensus readings in 46 patients. Visual adenosine perfusion CMR analysis revealed a higher sensitivity (87% versus 47%) but a lower specificity (71% versus 85%) of reader 1 compared to reader 2, while consensus readings yielded 73% sensitivity and 85% specificity. The observer dependency of stress perfusion CMR may limit the role of visual analysis of adenosine perfusion CMR for intermediate stenoses, as we have shown in the second study of this section.

Assessment of the relative myocardial perfusion index by stress perfusion CMR was also equally valid and reliable to FFR_CT_ to predict lesion-specific ischemia in a substudy with 39 intermediate-grade coronary stenoses. FFR value correlated equally strongly with FFR_CT_ (r = 0.675; p < 0.001) and the relative myocardial perfusion reserve index on stress perfusion CMR (r = –0.628; p < 0.001), while a poor correlation (r = 0.151; p = 0.36) was observed for the uncorrected perfusion analysis in culprit myocardium on stress perfusion CMR.

In conclusion, angiographic anatomical parameters using quantitative coronary CTA and coronary angiography poorly predict the functional significance of intermediate-grade stenosis. Functional assessment of intermediate-grade coronary stenosis by both stress perfusion CMR and FFR_CT_ allows strong correlation with invasive FFR measurement. Both techniques may be used as a safe non-invasive gatekeeper to guide patient selection for revascularization. Assessment of the functional significance of coronary stenoses estimated from stress perfusion CMR should be corrected for perfusion changes in remote myocardium to improve both the correlation with FFR and the diagnostic accuracy to predict FFR ≤ 0.80 lesions.
